# Supplementary material for: Development of a computer-aided design software for the quantitative evaluation of aesthetic damage
Source: PLoS One. 2019 Dec 18;14(12):e0226322. doi: 10.1371/journal.pone.0226322 (PMC6919621; doi:10.1371/journal.pone.0226322)
Supplement: S1 Code site — 1. (ZIP) [file pone.0226322.s001.zip › [EESC_jr][Projeto]Relatorio_07_21/reports/templates_zip/medialoot-lumino-admin-bootstrap-template.zip!/Lumino Admin Bootstrap Template/lumino/panels.html]

Lumino - Panels


Toggle navigation

LuminoAdmin

- User 
  - Profile
  - Settings
  - Logout

- Dashboard
- Widgets
- Charts
- Tables
- Forms
- Alerts & Panels
- Icons
- Dropdown
  - Sub Item 1
  - Sub Item 2
  - Sub Item 3
- Login Page

2. Icons

# Alerts & Panels

## Alerts

Welcome to the admin dashboard panel bootstrap template

Welcome to the admin dashboard panel bootstrap template

Welcome to the admin dashboard panel bootstrap template

Welcome to the admin dashboard panel bootstrap template

## Basic Panels

Default Panel

Lorem ipsum dolor sit amet, consectetur adipiscing elit. Pellentesque ut ante in sapien blandit luctus sed ut lacus. Phasellus urna est, faucibus nec ultrices placerat, feugiat et ligula. Donec vestibulum magna a dui pharetra molestie. Fusce et dui urna.

Primary Panel

Lorem ipsum dolor sit amet, consectetur adipiscing elit. Pellentesque ut ante in sapien blandit luctus sed ut lacus. Phasellus urna est, faucibus nec ultrices placerat, feugiat et ligula. Donec vestibulum magna a dui pharetra molestie. Fusce et dui urna.

Success Panel

Lorem ipsum dolor sit amet, consectetur adipiscing elit. Pellentesque ut ante in sapien blandit luctus sed ut lacus. Phasellus urna est, faucibus nec ultrices placerat, feugiat et ligula. Donec vestibulum magna a dui pharetra molestie. Fusce et dui urna.

Info Panel

Lorem ipsum dolor sit amet, consectetur adipiscing elit. Pellentesque ut ante in sapien blandit luctus sed ut lacus. Phasellus urna est, faucibus nec ultrices placerat, feugiat et ligula. Donec vestibulum magna a dui pharetra molestie. Fusce et dui urna.

Warning Panel

Lorem ipsum dolor sit amet, consectetur adipiscing elit. Pellentesque ut ante in sapien blandit luctus sed ut lacus. Phasellus urna est, faucibus nec ultrices placerat, feugiat et ligula. Donec vestibulum magna a dui pharetra molestie. Fusce et dui urna.

Danger Panel

Lorem ipsum dolor sit amet, consectetur adipiscing elit. Pellentesque ut ante in sapien blandit luctus sed ut lacus. Phasellus urna est, faucibus nec ultrices placerat, feugiat et ligula. Donec vestibulum magna a dui pharetra molestie. Fusce et dui urna.

## Widget Panels

Blue Panel

Lorem ipsum dolor sit amet, consectetur adipiscing elit. Pellentesque ut ante in sapien blandit luctus sed ut lacus. Phasellus urna est, faucibus nec ultrices placerat, feugiat et ligula. Donec vestibulum magna a dui pharetra molestie. Fusce et dui urna.

Teal Panel

Lorem ipsum dolor sit amet, consectetur adipiscing elit. Pellentesque ut ante in sapien blandit luctus sed ut lacus. Phasellus urna est, faucibus nec ultrices placerat, feugiat et ligula. Donec vestibulum magna a dui pharetra molestie. Fusce et dui urna.

Orange Panel

Lorem ipsum dolor sit amet, consectetur adipiscing elit. Pellentesque ut ante in sapien blandit luctus sed ut lacus. Phasellus urna est, faucibus nec ultrices placerat, feugiat et ligula. Donec vestibulum magna a dui pharetra molestie. Fusce et dui urna.

Red Panel

Lorem ipsum dolor sit amet, consectetur adipiscing elit. Pellentesque ut ante in sapien blandit luctus sed ut lacus. Phasellus urna est, faucibus nec ultrices placerat, feugiat et ligula. Donec vestibulum magna a dui pharetra molestie. Fusce et dui urna.

## Tab Panels

- Tab 1
- Tab 2
- Tab 3

#### Tab 1

Lorem ipsum dolor sit amet, consectetur adipiscing elit. Donec eget rutrum purus. Donec hendrerit ante ac metus sagittis elementum. Mauris feugiat nisl sit amet neque luctus, a tincidunt odio auctor.

#### Tab 2

Lorem ipsum dolor sit amet, consectetur adipiscing elit. Donec eget rutrum purus. Donec hendrerit ante ac metus sagittis elementum. Mauris feugiat nisl sit amet neque luctus, a tincidunt odio auctor.

#### Tab 3

Lorem ipsum dolor sit amet, consectetur adipiscing elit. Donec eget rutrum purus. Donec hendrerit ante ac metus sagittis elementum. Mauris feugiat nisl sit amet neque luctus, a tincidunt odio auctor.

- Tab 1
- Tab 2
- Tab 3

#### Tab 1

Lorem ipsum dolor sit amet, consectetur adipiscing elit. Donec eget rutrum purus. Donec hendrerit ante ac metus sagittis elementum. Mauris feugiat nisl sit amet neque luctus, a tincidunt odio auctor.

#### Tab 2

Lorem ipsum dolor sit amet, consectetur adipiscing elit. Donec eget rutrum purus. Donec hendrerit ante ac metus sagittis elementum. Mauris feugiat nisl sit amet neque luctus, a tincidunt odio auctor.

#### Tab 3

Lorem ipsum dolor sit amet, consectetur adipiscing elit. Donec eget rutrum purus. Donec hendrerit ante ac metus sagittis elementum. Mauris feugiat nisl sit amet neque luctus, a tincidunt odio auctor.
